# Deep phenotype unsupervised machine learning revealed the significance of pachychoroid features in etiology and visual prognosis of age-related macular degeneration

**DOI:** 10.1038/s41598-020-75451-5

**Published:** 2020-10-28

**Authors:** Yoshikatsu Hosoda, Masahiro Miyake, Kenji Yamashiro, Sotaro Ooto, Ayako Takahashi, Akio Oishi, Manabu Miyata, Akihito Uji, Yuki Muraoka, Akitaka Tsujikawa

**Affiliations:** 1 Department of Ophthalmology and Visual Sciences, Kyoto University Graduate School of Medicine, 54 Shogoin, Kawahara, Sakyo, Kyoto, 606-8507 Japan; 2 Department of Ophthalmology, Otsu Red-Cross Hospital, Otsu, Japan

**Keywords:** Macular degeneration, Retinal diseases, Classification and taxonomy, Machine learning

## Abstract

Unsupervised machine learning has received increased attention in clinical research because it allows researchers to identify novel and objective viewpoints for diseases with complex clinical characteristics. In this study, we applied a deep phenotyping method to classify Japanese patients with age-related macular degeneration (AMD), the leading cause of blindness in developed countries, showing high phenotypic heterogeneity. By applying unsupervised deep phenotype clustering, patients with AMD were classified into two groups. One of the groups had typical AMD features, whereas the other one showed the pachychoroid-related features that were recently identified as a potentially important factor in AMD pathogenesis. Based on these results, a scoring system for classification was established; a higher score was significantly associated with a rapid improvement in visual acuity after specific treatment. This needs to be validated in other datasets in the future. In conclusion, the current study demonstrates the usefulness of unsupervised classification and provides important knowledge for future AMD studies.

## Introduction

Age-related macular degeneration (AMD) is the leading cause of blindness in developed countries^1,2^. For the development of AMD, inflammation of the retinal pigment epithelium (RPE) and drusen are thought to play an important role^3–6^. AMD is often associated with neovascularization of the choroid and is then called neovascular AMD, which can be treated by anti-VEGF agents such as aflibercept and ranibizumab.

Neovascular AMD includes different types of neovascularization, the most common being choroidal neovascularization (CNV). CNV refers to neovascularization from the choroid through a break in the Bruch’s membrane into the sub-RPE or subretinal space. The one that is limited within the sub-RPE space is called type 1 CNV, whereas that which grows into the subretinal space is called type 2 CNV. Polypoidal choroidal vasculopathy (PCV) is characterized by a branching network of inner choroidal vessels with terminal polypoidal lesions that progress to hemorrhagic complication. While PCV was originally reported as a distinct clinical entity, it is currently considered to be a variation of type 1 CNV^7–12^. Retinal angiomatous proliferation (RAP) is also recognized as a subtype of AMD^13^. RAP begins as a proliferation of vessels within the retina, which merge with the choroidal circulation in the late stage. Because its origin is not the choroid, RAP has been recently described as type 3 neovascularization, and not CNV.

While all these subcategories of AMD are observed in both Asians and Caucasians, their clinical characteristics differ between the two populations. For example, PCV is more prevalent in the Asian than in the Caucasian population (23–49% in Asian vs 8–9% in Caucasian)^14–19^, while RAP prevalence shows the opposite pattern (5–11% in Asian vs 15–20% in Caucasian)^20–26^. Typically, soft confluent drusen and pseudodrusen are less observed in Asian AMD individuals^27,28^, which is in contrast to the higher prevalence of choroidal vascular hyperpermeability (CVH) and thickened choroid in these individuals^29–31^.

With regard to this heterogeneity between Asian and Caucasian AMD populations, a newly proposed clinical manifestation named “pachychoroid” has emerged as an important aspect of CNV^32,33^. Although there are no definite criteria, “pachychoroid” is diagnosed when a combination of pachychoroid-related features such as thickened choroid, CVH, dilated choroidal vessels (pachyvessels), reduced fundus tessellation, and pachydrusen is observed. Although the term pachychoroid neovasculopathy (PNV), which refers to CNV of pachychoroid etiology, was first used in 2015^34^, type 1 CNV with pachychoroid-related features is known to masquerade as neovascular AMD^35^. In 2015, we reported that some proportion of Japanese AMD might be PNV^36^. Considering that several studies, including ours, have suggested that AMD with thickened choroid shows a different response to photodynamic therapy or relatively good visual prognosis after anti-VEGF therapy compared to that without thickened choroid^29,31,36–41^, pachychoroid-related features should definitely be considered in AMD management.

There is high heterogeneity within the CNV subtypes, suggesting a need for recategorization of CNV based on clinical manifestations, including pachychoroid-related features, to confirm the validity of the traditional subcategorization. In the current study, we applied a deep phenotype machine learning method, which is a comprehensive analysis of phenotypic abnormalities, to classify patients with unilateral CNV using all AMD- and pachychoroid-related features. Unsupervised machine learning algorithm was applied to obtain objective, data-driven CNV classification.

## Methods

The current study was approved by the Institutional Review Board at Kyoto University Graduate School of Medicine and adhered to the tenets of the Declaration of Helsinki. Written informed consent was obtained from each patient. The participants are registered under the Cohort Study of the Clinical Course of Macular Diseases in Japanese Patients (ClinicalTrials.gov identifier: NCT02081339).

### Participants

We reviewed the medical records of consecutive patients (1) who had visited the macular service of Kyoto University Hospital (Kyoto, Japan) between January 2012 and July 2018, (2) who agreed to participate in the study, and (3) who were diagnosed with unilateral choroidal neovascularization (diagnostic criteria are described in the following section).

Patients with the following conditions were excluded from the study: (1) myopic CNV, CNV secondary to trauma, angioid streaks, uveitis, or any other neovascular maculopathy; (2) patients with history of retinal angiomatous proliferation (RAP), epiretinal membrane, retinal vein occlusion or any other macular diseases except for CSC in either eye; (3) those for whom the data of ophthalmic evaluation was lacking: (4) those with history of ocular surgery except for cataract surgery: and (5) those with history of CNV treatment.

History of CSC, hypertension, diabetes mellitus, hyperlipidemia, cerebral infarction, and myocardial infarction was obtained through a medical interview. Smoking status was also established and converted to the Brinkman Index ([number of cigarettes per day] × [number of years smoking]).

### Multimodal imaging

All participants underwent complete ophthalmological examinations, including best-corrected visual acuity (BCVA) assessment, axial length (AL) measurement (IOL Master, Carl Zeiss, Germany), and slit-lamp examination. After pupil dilation, Color fundus photographs (field, 40°) were obtained from all participants digitally using a Topcon TRC NW6S non-mydriatic retinal camera (Topcon, Tokyo, Japan).

Infrared reflectance (IR), fundus autofluorescence (FAF), fluorescein angiography (FA), and indocyanine green angiography (ICGA) images were acquired using a confocal scanning laser ophthalmoscope (SLO) (Spectralis HRA + OCT; Heidelberg Engineering, Heidelberg, Germany). The field of view was set to 30° × 30° centered on the macula.

Spectral-domain optical coherence tomography (SD-OCT) was conducted using a Spectralis HRA + OCT (Heidelberg Engineering, Heidelberg, Germany). First, horizontal and vertical line scans through the fovea center were obtained at a 30° angle, followed by serial horizontal scans with an examination field size of 30° × 10°. Inverted OCT images, used for measuring choroidal thickness, were routinely obtained in all patients using an EDI technique. In patients who lacked AL data at baseline, the results of subsequent measurements were used for analysis.

### Image analysis and phenotyping

For the subsequent unsupervised machine learning, we phenotyped a total of 61 baseline characteristics. All parameters evaluated are shown in Supplementary Table 1.

**Figure 1:**
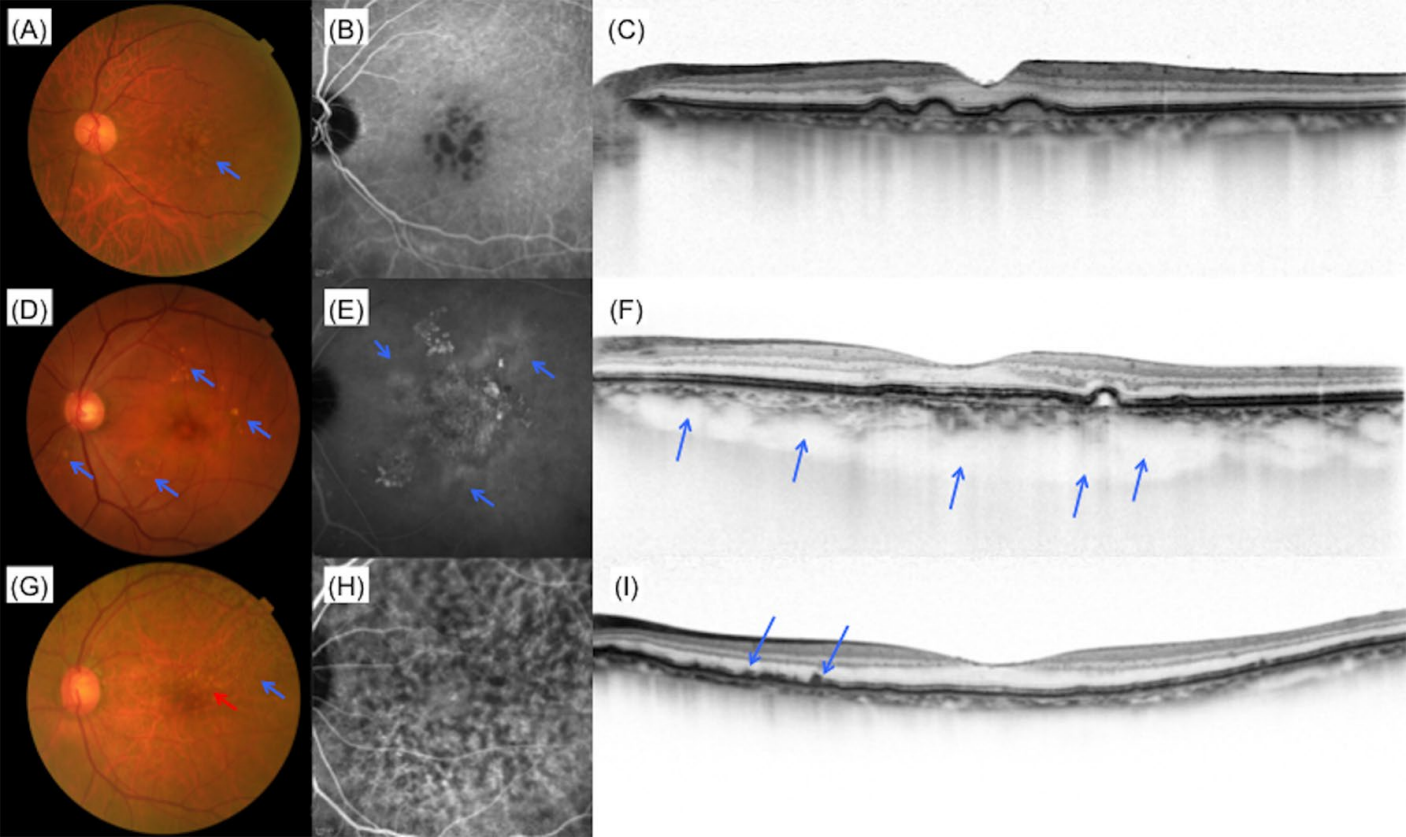
Examples of clinical characteristics. (**A**–**C**) Images from CNV fellow eye of a 79 year old male patient. (**A**) Color fundus photograph. Type A drusen in the macular region (blue arrow). (**B**) Late phase of ICGA image. (**C**) Foveal horizontal OCT scan shows choroidal thinning. (**D**–**F**) Images from CNV fellow eye of a 64 year old male patient. (**D**) Color fundus photograph shows multiple type B and C drusen within and outside macular region (blue arrows). (**E**) Choroidal hyperpermeability in late phase of ICGA (blue arrows). (**F**) Foveal horizontal EDI OCT scan shows choroidal thickening and dilated choroidal vessels (blue arrows). (**G**–**I**) Images from CNV fellow eye of an 87 year old female patient. (**G**) Color fundus photograph shows type A drusen (red arrow) and pseudodrusen (blue arrow) within macular region. (**H**) Late phase of ICGA. (**I**) Foveal horizontal OCT scan shows the accumulation of deposits on retinal pigment epithelium (blue arrows). *CNV* choroidal neovascularization, *EDI* enhanced depth imaging, *ICGA* indocyanine green angiography, *OCT* optical coherence tomography.

The type of extracellular deposit (drusen) was determined based on color fundus photographs. Eyes without drusen or with small drusen (< 63 μm) were considered as not presenting significant drusen. Each druse was categorized into 3 types based on the fundus photographs according to the Spaide’s report on soft drusen and pachychoroid-associated drusen^38^. Briefly, conventional soft drusen that aggregated in the central macula under the subRPE, with a poorly defined ovoid outer contour were labeled as “Type A drusen” (Fig. 1A,C). Drusen with a round or ovoid shape and a more complex outer contour were labeled as “Type B drusen,” while the drusen with an undercut and eroded outer contour were labeled as “Type C drusen” (Fig. 1D). Drusen that aggregated within 1-disc diameter were considered as a single drusen cluster. For each category of drusen, we evaluated the size of cluster (measured using increments of 0.5 disc size), the number of clusters within macular region, and the number of clusters located outside macular region, for the right eye and left eye, respectively. The presence of dot- or ribbon-type pseudodrusen was confirmed using color fundus photography, IR, FAF, ICGA (late phase), or in the cases with OCT evidence of definite drusenoid deposits above the RPE. The examples of fundus photograph with these types of drusen are shown in Fig. 1G–I.

Subfoveal choroidal thickness (SFCT) was defined as the vertical distance between Bruch’s membrane and the chorioscleral interface at the fovea and was manually measured on horizontal EDI-OCT images. Central retinal thickness (RT) was defined as the vertical distance between inner limiting membrane and Bruch’s membrane, and was manually measured on foveal OCT images. Lesion size was determined as the greatest linear dimension (GLD) measured with fluorescein angiography examination before treatment. The GLD was determined as a lesion with any classic or occult CNV in eyes with AMD. When a contiguous retinal pigment epithelium (RPE) detachment or subretinal hemorrhage was observed, it was included in the lesion according to the Treatment of age-related macular degeneration with photodynamic therapy Study (TAP study) protocol^42^. Presence of polypoidal lesions, polypoidal structures at the border of the branching vascular network, was investigated on ICG angiograms. The type of CNV was also evaluated; type 1 CNV represents CNV that locate beneath the RPE, and type 2 CNV represents CNV penetrating the RPE.

Choroidal vascular hyperpermeability (CVH) was determined by investigating the presence of multifocal hyperfluorescent areas with blurred margins that expanded during the late phase of ICGA (i.e., 10–15 min after dye injection) (Fig. 1B,E). Dilated choroidal vessels (pachyvessel) were defined as those that retain a large caliber and occupy a significant proportion of the total choroidal volume, and present a relative thinning of the choriocapillaris layer in line or serial OCT images as previously reported (Fig. 1F). Reduced fundus tessellation was defined as a focal, multifocal, or diffuse area of reddish orange background within the arcades with minimal to absent choroidal vascular markings on color fundus photographs. All the protocols used in the phenotyping of baseline characteristics are shown in Supplementary Table 2. Under the supervision of macular disease specialist (M.M.), another macular disease specialist (Y.H.) evaluated these characteristics. If there were any doubt about the phenotypes, M.M. and Y.H. would discuss it face to face and judge the imaging features.

### Automatic categorization of patients with CNV using machine learning

Cluster analysis is an established and pivotal method for unsupervised machine learning (ML). In particular, K-means procedure is a widely accepted unsupervised machine learning algorithm, which uses centroids to represent clusters^43,44^. After the number of clusters is chosen, every data point is allocated to one of the clusters by minimizing the within-cluster sum of squares. Previous studies have demonstrated the usefulness of the K-means method for classifying a phenotypically heterogeneous heart failure cohort based on patients’ characteristics, which enabled to identify a cluster of responders to specific therapies^45–47^. Before applying K-means method, principal component analysis (PCA) is usually applied for transforming a number of correlated variables into a number of uncorrelated variables^48,49^. However, determining the optimal number of clusters during application of K-means method was considered as a major difficulty^50^. As a solution, gap statistical analysis, an algorism for estimating the optimal number of clusters by comparing the change in within-cluster dispersion with that expected under an appropriate reference null distribution, was introduced in 2002^51^.

To automatically classify patients with CNV, we applied k-means method. First, PCA was performed using 61 baseline characteristics after standardization. To calculate the validity of clustering measure, we used gap statistics analysis. Briefly, we calculated the sum of the pairwise distance for all points (W_k_) upon clustering the dataset into *k* clusters. Then, we standardized the log (W_k_) graph by comparing its expectation under an appropriate null reference distribution of the data. The best number of clusters was defined as the smallest *k* such that its value of compactness was not more than 1 standard error away from the first local maximum. We then performed k-means clustering using all 60 principal components to classify all patients with CNV.

### CNV scoring system

We divided the participants into two datasets, a training set, and a validation set, by the ratio of 3 to 1, using computerized randomization. Four hundred and two eyes (74.9%) within the study population were selected as the training set and 135 eyes (25.1%) as the validation set.

Using the training set, we designed a new scoring system to distinguish the groups. We performed logistic regression analysis for the clusters that were automatically determined by k-means method. In this logistic regression analysis, we used parameters based on the results. Details of parameter selection are shown in the Result section. Based on the result of logistic regression analysis, we established CNV scoring system and calculated the CNV score for each participant. The diagnostic ability of the scoring system was validated using the validation dataset.

### Association of the score with the clinical outcome

To evaluate the clinical significance of the score, we assessed the association between the score and the treatment outcome. Patients treated with intravitreal aflibercept (IVA) injection by fixed regimen (i.e. 3 times of monthly IVA followed by 4 times bimonthly IVA during 12 months from the initial treatment) from July 2012 to August 2017 were included in this analysis.

We conducted analyses; namely, a linear regression analysis to evaluate the association between CNV score and the change of logMAR from baseline at 3 and 12 months after initial treatment adjusting for age, sex, and baseline logMAR. Patients with no follow-up visit were excluded, and we applied last observation carried forward (LOCF) imputation to cope with selection bias^52–56^.

### Statistical analyses

All BCVA measurements were converted to logMAR equivalents before analyses. Every 2 × 2 table was evaluated using Fisher’s exact test. Continuous variables were compared using the Wilcoxon test. A P-value < 0.05 was considered statistically significant. These statistical analyses were conducted using R software ver.3.5.2 (https://www.r-project.org/) and JMP 13 (SAS Institute Inc., Cary, NC, USA). For the gap statistics analysis, the CRAN package “clusGap” was used.

## Results

### Participants and background

Six hundred and seventeen consecutive patients were considered for the present study. Seventy-two patients were excluded due to lack of data; specifically, 53 patients lacked axial length data, because axial length was not routinely measured before April 2013; 10 patients lacked FAF/ICGA examination, because of drug allergy; 6 patients lacked OCT images measured by Spectralis; 3 patients lacked information on internal medicine, treatment, or smoking history. Moreover, we excluded 5 patients who lacked choroidal thickness data due to poor EDI OCT images; one patient whose GLD could not be measured due to poor images; and 3 patients whose CNV types were not evaluated due to severe fibrosis. Therefore, a total of 537 patients were finally included in the study (Supplementary Figure 1). Extracts of baseline characteristics are presented in Table 1. Complete patient information is shown in Supplementary Table 1.

**Table 1:** Baseline characteristics of the participants.

Number of patients	537
Male (%)	387 (72.1%)
Female (%)	150 (27.9%)
Age (years)	73.11 ± 8.43
History of CSC (%)	28 (5.2%)
LogMAR BCVA in CNV-affected eye	0.322 ± 0.363
Axial length in CNV-affected eye (mm)	23.62 ± 1.09
Retinal thickness in CNV-affected eye (μm)	402.80 ± 208.10
Choroidal thickness in CNV-affected eye (μm)	259.15 ± 108.94
Choroidal thickness in fellow eye (μm)	255.94 ± 104.79
CVH (+) in either eye (%)	125 (23.3%)
Polypoidal region (+) in CNV-affected eye	254 (47.3%)
Choroidal vessel dilation (+) in either eye	293 (54.6%)
Reduced fundus tessellation (+) in either eye	233 (43.4%)
Pseudodrusen (+) in either eye	29 (5.4%)
Type A drusen (+) in either eye	93 (17.3%)
Type B or C drusen (+) in either eye	337 (62.8%)

All values are shown as mean ± standard deviation and compared using Wilcoxon test. Fisher’s exact test was used to evaluate every 2 × 2 table.

*CSC* central serous chorioretinopathy, *MAR* minimum angle resolution, *BCVA* best-corrected visual acuity, *CNV* choroidal neovascularization, *CVH* choroidal vascular hyperpermeability.

### Clustering CNV patients using ML

Gap statistical analysis determined that the optimal number of clusters was two, suggesting that dividing CNV patients into two groups is most appropriate. The standardized log (W_k_), its expectation value under an appropriate null reference distribution (E.log (W_k_)), the gap between these 2 values, and the standard error of log (W_k_) are summarized in Supplementary Table 3. The line graph of gap and cluster number is shown in Supplementary Figure 2.

**Figure 2:**
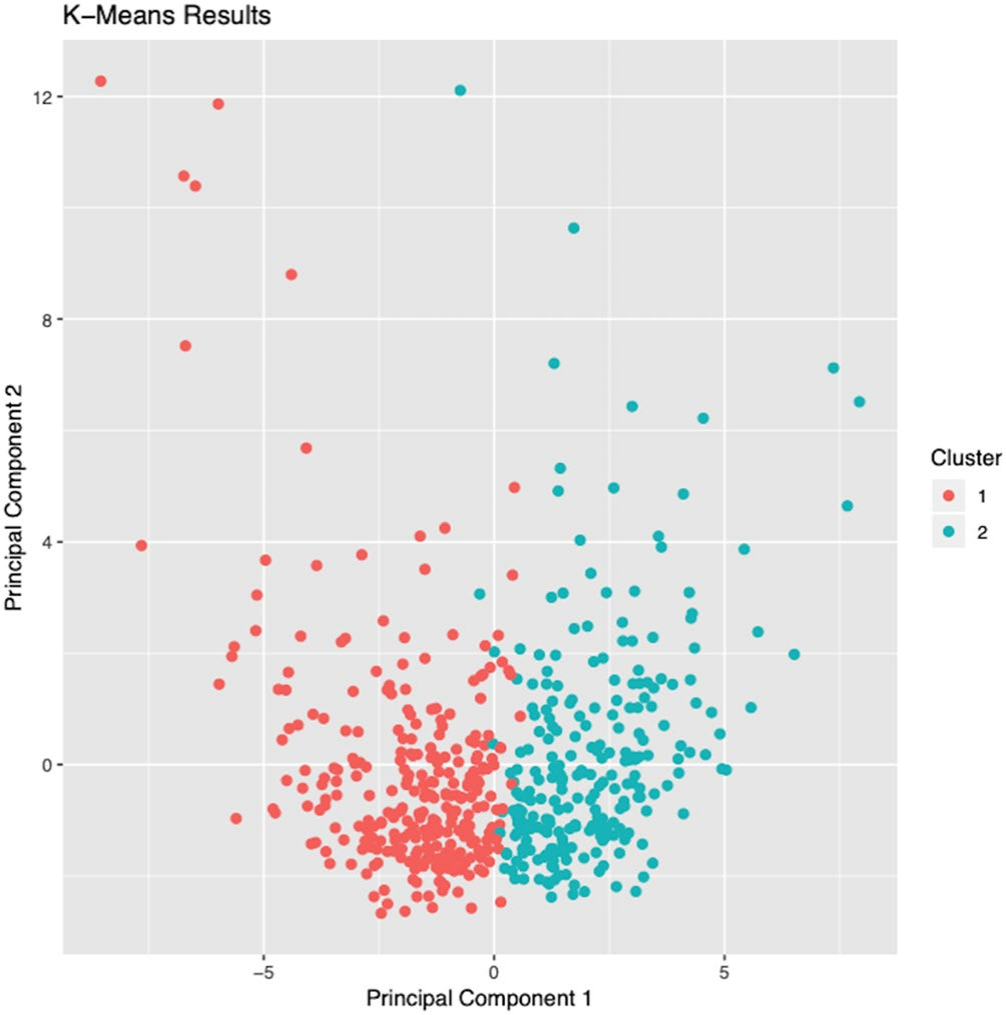
Scatter plot showing the result of machine learning clustering. Unsupervised machine learning algorithm was used to cluster patient data. The scatter plot indicates the position of the subjects according to the similarity by dimensionality reduction. Patients were divided into cluster 1 (red dots, N = 289) and cluster 2 (blue dots, N = 247).

By applying k-means analysis, patients with CNV were divided into 2 clusters, cluster 1 (289 patients, 53.8%) and cluster 2 (248 patients, 46.2%). Scatter plot of principal component 1 vs principal component 2 is shown in Fig. 2, with cluster 1 as represented as red dots and cluster 2 as blue dots.

### Differences in characteristics between the two clusters

**Figure 3:**
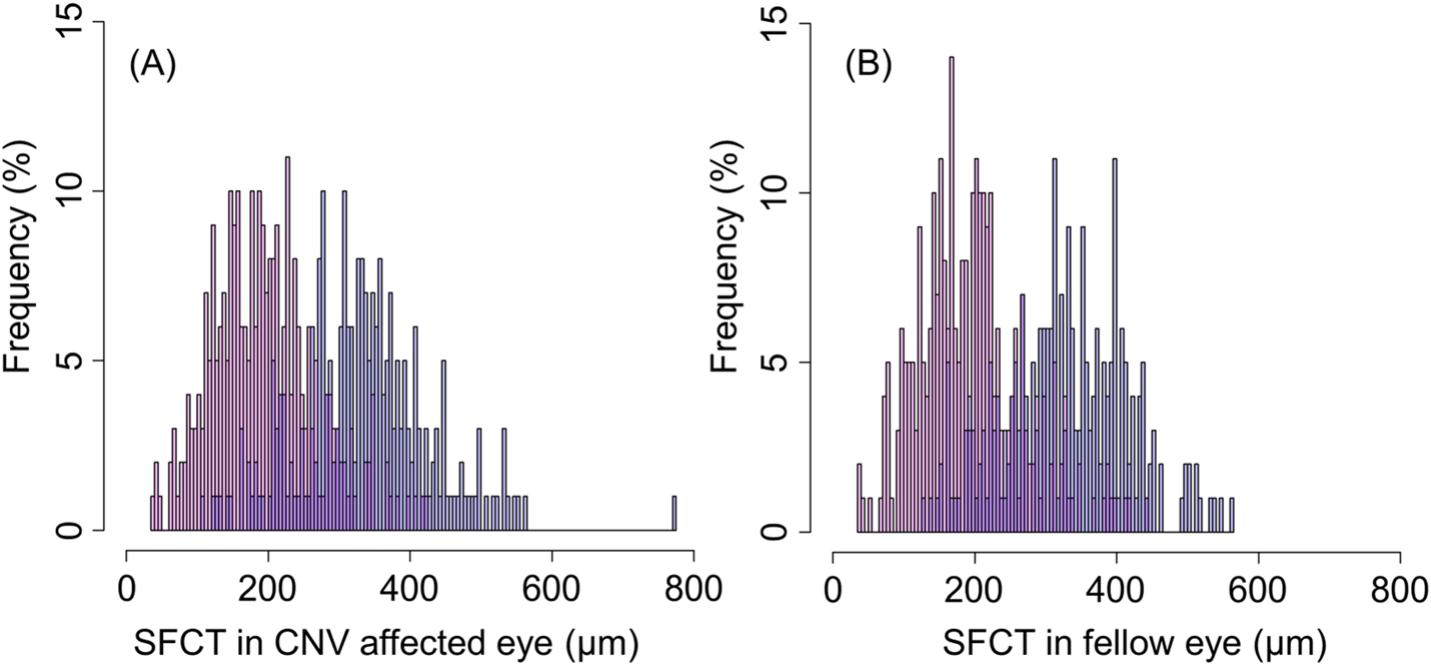
Histograms of subfoveal choroidal thickness in CNV-affected eye (**A**) and fellow eye (**B**). Superposed histograms of patients in PNV-type cluster (blue) and AMD-type cluster (red) show binominal distribution of subfoveal choroidal thickness (SFCT) in both eyes. CNV; choroidal neovascularization.

Differences in characteristics between the two clusters are summarized in Table 2. Patients in cluster 1 were older than the patients in cluster 2 (75.0 ± 7.9 and 70.9 ± 8.5, P < 0.0001). The patients in cluster 2 showed higher frequency of CVH, dilated choroidal vessel and reduced fundus tessellation, thicker choroid, and better BCVA in both eyes compared to patients in cluster 1. Histograms of SFCT in CNV-affected eyes and fellow eyes are shown in Fig. 3, demonstrating a clear bimodal distribution.

**Table 2:** Summary of differences in baseline characteristics between 2 groups.

	Cluster 1 (AMD-type)	Cluster 2 (PNV-type)	*P* value*
Number of patients	289	248	
Male (%)	208 (72.0%)	179 (72.2%)	
Female (%)	81 (28.0%)	69 (27.8%)	1.000
Age (years)	75.03 ± 7.87	70.87 ± 8.53	< 0.0001
History of CSC (%)	4 (1.4%)	24 (9.7%)	< 0.0001
LogMAR BCVA in CNV-affected eye	0.377 ± 0.385	0.259 ± 0.324	0.0001
Axial length in CNV-affected eye (mm)	23.83 ± 1.10	23.37 ± 1.01	< 0.0001
Axial length in fellow eye (mm)	23.97 ± 1.13	23.46 ± 1.02	< 0.0001
Retinal thickness in CNV-affected eye (μm)	451.46 ± 230.42	346.11 ± 161.47	< 0.0001
Choroidal thickness in CNV-affected eye (μm)	194.56 ± 74.19	334.42 ± 93.65	< 0.0001
Choroidal thickness in fellow eye (μm)	194.24 ± 74.29	327.85 ± 88.01	< 0.0001
CVH (+) in either eye (%)	18 (6.2%)	107 (43.1%)	< 0.0001
Polypoidal region (+) in CNV-affected eye	127 (43.9%)	127 (51.2%)	0.0999
Choroidal vessel dilation (+) in either eye	80 (27.7%)	213 (85.9%)	< 0.0001
Reduced fundus tessellation (+) in either eye	33 (15.9%)	200 (80.6%)	< 0.0001
Pseudodrusen (+) in either eye	28 (9.7%)	1 (0.4%)	< 0.0001
Type A drusen (+) in either eye	82 (28.4%)	11 (4.4%)	< 0.0001
Type B drusen (+) in either eye	107 (37.0%)	177 (71.4%)	< 0.0001
Type C drusen (+) in either eye	56 (19.4%)	122 (49.2%)	< 0.0001
Type B or C drusen (+) in either eye	133 (46.0%)	204 (82.3%)	< 0.0001

All values are shown as mean ± standard deviation and compared using Wilcoxon test. Fisher’s exact test was used to evaluate every 2 × 2 table.

*AMD* age-related macular degeneration, *PNV* pachychoroid neovasculopathy, *CSC* central serous chorioretinopathy, *MAR* minimum angle resolution, *BCVA* best-corrected visual acuity, *CNV* choroidal neovascularization, *CVH* choroidal vascular hyperpermeability.

The frequency of pseudodrusen and soft drusen (“type A drusen”) were higher in cluster 1 patients, while the frequency of type B and C drusen were higher in cluster 2 patients. Although there was no significant difference in the frequency of type 1 CNV, that of type 2 CNV was higher in cluster 1 patients (*P* = 0.0097). Retinal thickness in CNV-affected eyes was significantly greater in cluster 1 patients compared to cluster 2 patients (*P* < 0.0001).

Complete results of the comparisons between the 2 clusters are shown in Supplementary Table 1. Out of the total 537 patients, 219 patients met our previous PNV diagnostic criteria^36^. Among these 219 patients, 195 patients (89%) were classified into cluster 2 by K-means algorithm. From these results, we describe cluster 1 as AMD-type cluster and cluster 2 as PNV-type cluster.

However, 24 patients who met our previous PNV diagnostic criteria were classified into AMD-type cluster. The fundus photographs, OCT, and ICGA images from patients who meet our previous PNV diagnostic criteria and belong to AMD-type cluster are shown in Supplementary Figure 3. We found that these patients are borderline cases between PNV and AMD.

### CNV scoring system

As machine learning classified CNV patients into PNV-type and AMD-type patients, we established CNV scoring system using 7 traditionally-defined AMD-associated parameters, namely, age, sex, central retinal thickness in CNV-affected eye, SFCT in the CNV-affected eye, SFCT in the fellow eye, the presence of CVH in either eye, and the presence of soft drusen (“type A drusen”) in either eye. Based on the logistic regression analysis, the scoring model was established as shown in Table 3. The accuracy of this model was 0.91 (sensitivity and specificity were 0.844 and 0.972, respectively) and AUC was 0.939 in validation datasets. ROC curve of this model for validation dataset is shown in Supplementary Figure 4A. The higher CNV score indicates the higher probability of belonging to PNV-type cluster. Cutoff values to show negative predictive value of 100% and positive predictive value of 100% were − 3.610 and 2.629 (Table 4). The CNV score of all participants ranged from − 8.519 to 9.871.

**Table 3:** Logistic regression analysis on the results of clustering, and CNV scoring system.

	OR (95% CI)*	*P* value**	Score
Intercept	–	–	− 7.04
Age (every 10 years)	1.18 (0.79–1.76)	0.42	0.16
Sex (male: 0, female: 1)	1.67 (0.81–3.50)	0.17	0.52
CVH in either eye (+: 1, −: 0)	7.35 (3.23–17.89)	4.39 × 10^–6^	1.99
Retinal thickness in CNV-affected eye (every 100 μm)	0.71 (0.59–0.85)	2.07 × 10^–4^	− 0.34
Choroidal thickness in CNV-affected eye (every 100 μm)	3.91 (2.35–6.78)	4.25 × 10^–7^	1.37
Choroidal thickness in fellow eye (every 100 μm)	3.45 (2.08–5.90)	3.12 × 10^–6^	1.24
Type A drusen in either eye (+: 1, −: 0)	0.19 (0.059–0.53)	2.50 × 10^–3^	− 1.68

*CVH* choroidal vascular hyperpermeability, *CNV* choroidal neovascularization, *OR* odds ratio, *CI* confidence interval, *PNV* pachychoroid neovasculopathy, *AMD* age related macular degeneration.

*Odds ratio of PNV compared with AMD.

**P value was derived using logistic regression analysis on the clustering result.

**Figure 4:**
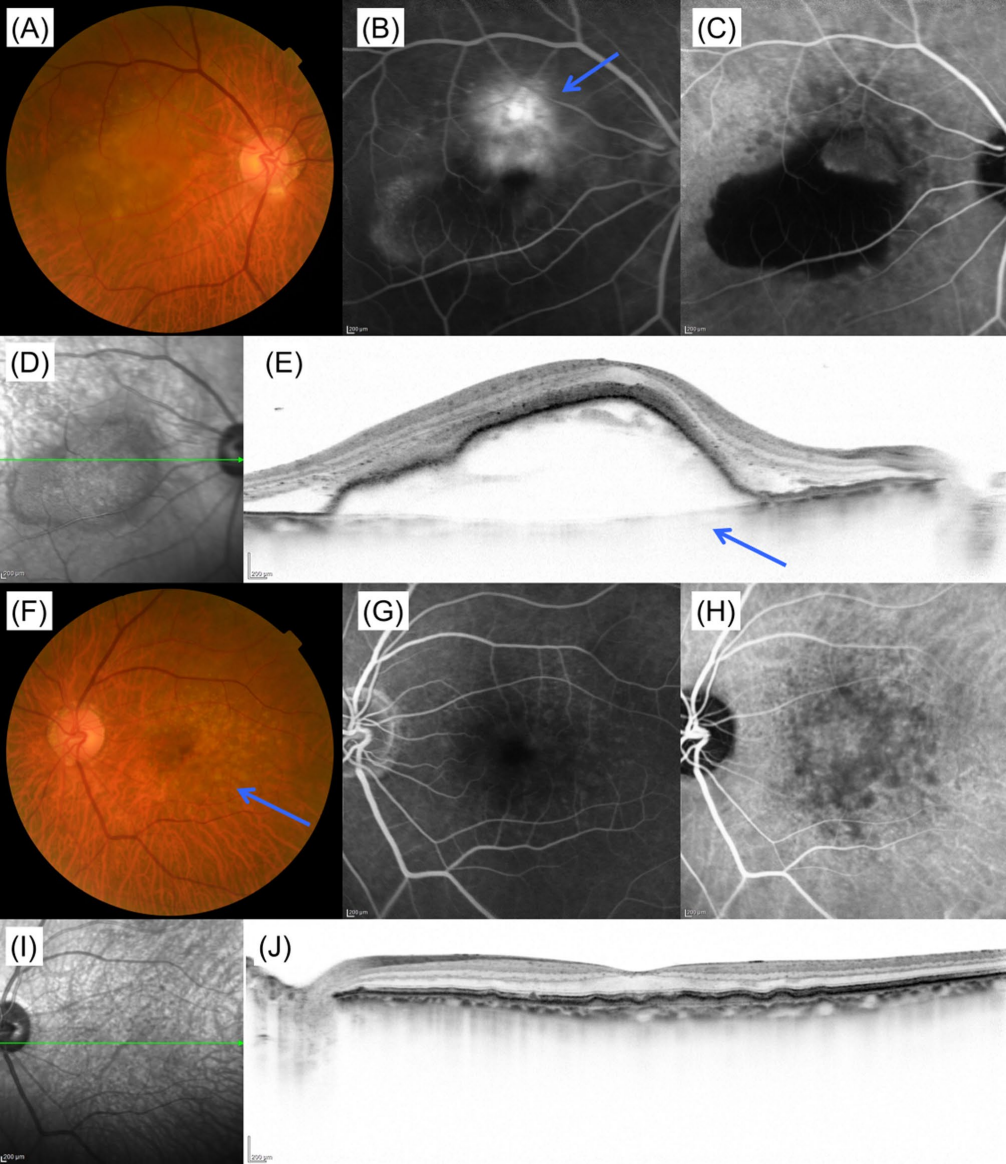
A case of low CNV score corresponding to AMD-type patient. A 76-year-old male patient was visually impaired in the right eye. Images from the right eye (**A**–**D**) and left eye (**E**–**I**). (**A**) Color fundus photograph shows large retinal pigment epithelium detachment. (**B**) Fluorescein angiography showed occult CNV suggesting type 1 CNV. (**C**) Late phase of indocyanine green angiography image shows no choroidal vascular hyperpermeability. (**D**) Infrared reflectance image of right eye. (**E**) Foveal horizontal OCT scan shows large pigment epithelial detachment consistent with CNV lesion. Subfoveal choroidal thickness was 128 μm and retinal thickness was 1197 μm. (**F**) Color fundus photograph shows type A drusen within macular lesion (blue arrow). There are no type B drusen or type C drusen. (**G**) Fluorescein angiography image suggests no CNV. (**H**) Late phase of indocyanine green angiography image shows no choroidal vascular hyperpermeability. (**I**) Infrared reflectance image of left eye. (**J**) Foveal horizontal OCT scan shows a subretinal deposit consistent with type A drusen. Subfoveal choroidal thickness was 145 μm and retinal thickness was 252 μm. CNV score of this patient was − 7.978, indicating AMD. *CNV* choroidal neovascularization, *OCT* optical coherence tomography, *AMD* age-related macular degeneration.

**Table 4:** Positive predictive ratio and CNV score cutoff in validation dataset.

	< − 3.610	− 3.610 to − 1.966	− 1.967 to 0.697	0.697 to 2.629	2.629 <
Cluster 1 (AMD-type)	21	19	29	2	0
Cluster 2 (PNV-type)	0	2	8	25	29
Positive predictive value of PNV (positive predictive value of AMD)	(100%)	(90.5%)	(78.4%)	92.6%	100%

*AMD* age-related macular degeneration, *PNV* pachychoroid neovasculopathy, *CNV* choroidal neovascularization.

**Figure 5:**
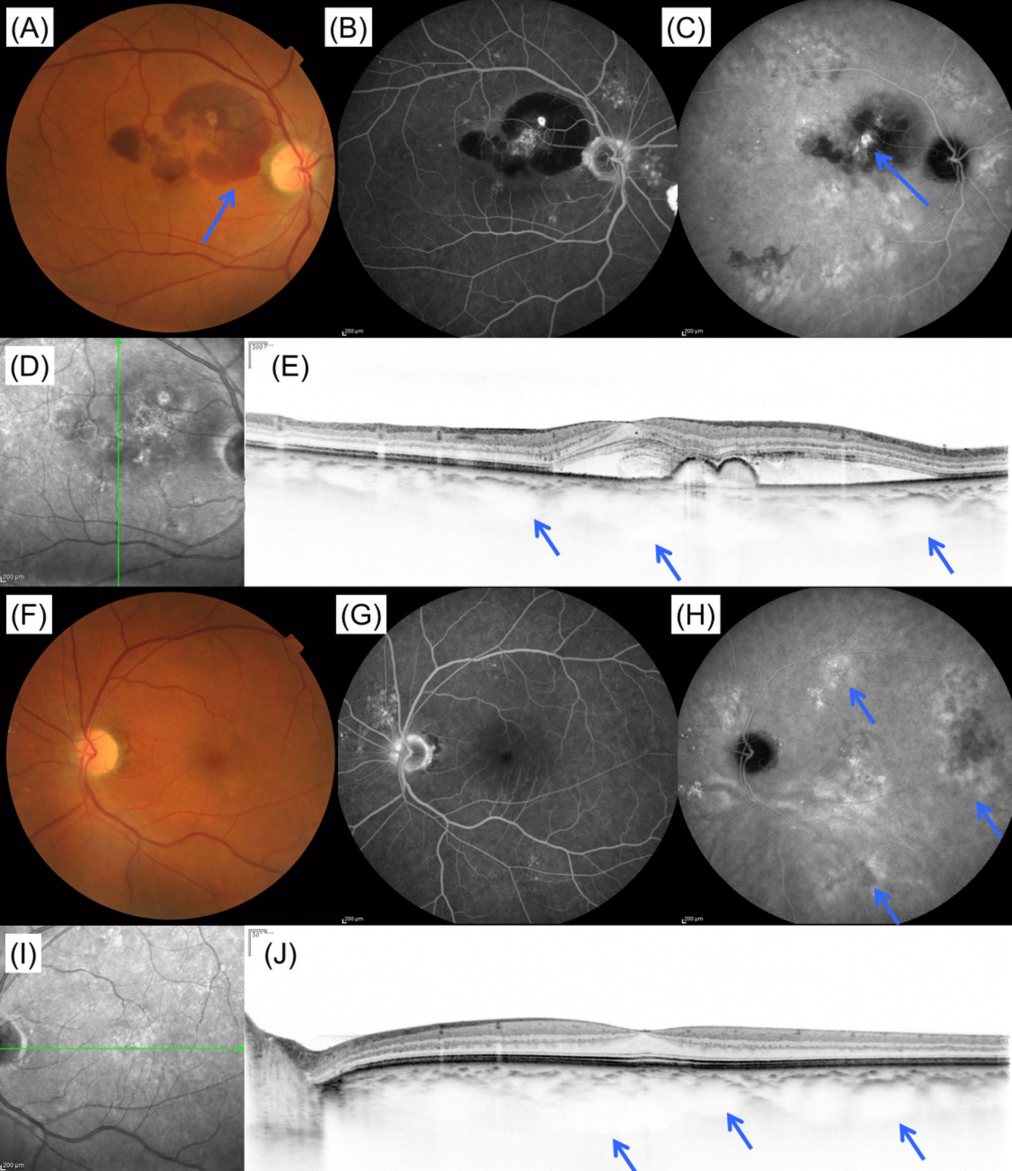
A case of high CNV score corresponding to PNV-type patient. A 66-year-old male patient was visually impaired in the right eye. Images from the right eye (**A**–**D**) and left eye (**E**–**I**). (**A**) Color fundus photograph shows subretinal hemorrhage within macular region (blue arrow). (**B**) Fluorescein angiography image shows leakage within the region of subretinal hemorrhage. (**C**) Late phase of indocyanine green angiography image shows a polypoidal lesion (blue arrow). Choroidal vascular hyperpermeability spots were also observed around the macular region. (**D**) Infrared reflectance image of right eye. (**E**) Foveal vertical EDI OCT scan shows diffusely thickened choroid and pigment epithelial detachment. Dilated choroidal vessels were also observed (blue arrows). Subfoveal choroidal thickness was 508 μm and retinal thickness was 455 μm. (**F**) Color fundus photograph shows no CNV. (**G**) Fluorescein angiography image shows no leakage. (**H**) Late phase of indocyanine green angiography image shows multiple choroidal vascular hyperpermeability spots (blue arrows). (**I**) Infrared reflectance image of left eye. (**J**) Foveal horizontal EDI OCT scan shows diffusely thickened choroid and dilated choroidal vessels (blue arrows). Subfoveal choroidal thickness was 503 μm and retinal thickness was 262 μm. CNV score for this patient was 7.656, which indicating PNV. *CNV* choroidal neovascularization, *EDI* enhanced depth imaging, *OCT* optical coherence tomography, *PNV* pachychoroid neovasculopathy.

The fundus photographs, OCT and ICGA images from patient who belong to AMD-type cluster (CNV score = − 7.978) are shown in Fig. 4, while those from patient who belong to PNV-type cluster (CNV score = 7.656) are shown in Fig. 5.

### Association of CNV score with the clinical outcome

Of the 537 participants, 437 patients visited the hospital at least once, excluding the initial visit. Two-hundred thirty three of the 437 patients were treated with IVA injection by fixed regimen and eligible to evaluate the clinical outcome. LOCF imputation of visual acuity was applied for 3 patients at 3 months, 7 patients, and at 12 months. The linear regression analysis showed that, after adjusting for age, sex, and baseline visual acuity, a higher CNV score (i.e. increased probability of PNV) was significantly associated with a higher visual acuity improvement in CNV-affected eyes at 3 months (N_3months_ = 231, *P*_3months_ = 0.0044; Table 5), while there was no significant association between CNV score and visual acuity improvement at 12 months (N_12months_ = 236, *P*_12months_ = 0.056; Table 5). Time course of the changes in visual acuity for PNV-type cluster and AMD-type cluster are shown in Supplementary Figure 5.

**Table 5:** Association of CNV scores with changes in visual acuity at 3 and 12 months.

	No adjustment		Adjusted*	
	B (SE)	*P* value	B (SE)	*P* value
LogMAR BCVA change at 3 months	− 0.011 (0.0039)	0.0049	− 0.012 (0.0040)	0.0044
LogMAR BCVA change at 12 months	− 0.0065 (0.0050)	0.199	− 0.0096 (0.0050)	0.056

Linear regression analysis was performed to derive the *P* value.

*SE* standard error, *MAR* minimum angle resolution, *BCVA* best-corrected visual acuity.

*Adjusted for age, sex, and baseline logMAR BCVA.

## Discussion

In the present study, we applied unsupervised machine learning to automatically classify patients with CNV based on their clinical manifestation. This analysis suggested that dividing CNV patients into two groups is the most appropriate. The comparison of clinical characteristics between the two clusters indicated that one cluster included eyes with characteristics of PNV and the other cluster included eyes with characteristics of AMD. The CNV score obtained based on logistic regression results showed the possibility of diagnosing PNV and was significantly associated with visual prognosis at 3 months after anti-VEGF treatment.

The emergence of the concept of pachychoroid disease^34,36^ has led to an increased numbers of reports addressing this condition. However, as Spaide pointed, no golden standard diagnostic criteria has been established^38^, so most authors applied their own original diagnostic criteria which were lacking in scientific validation. In a clinical setting, clinicians diagnose pachychoroid spectrum based on a combination of clinical features, that include dilated choroidal vessels, reduced fundus tessellation, or pachydrusen, rather than a single one. In the current study, our data-driven approach, i.e. unsupervised machine learning using all pachychoroid-related and AMD-related characteristics^36,38,57^, successfully divided CNV into those with pachychoroid spectrum versus those representing conventional typical AMD. Because 89% of the patients who met our previous, tentative PNV criteria were classified into cluster 2, it is plausible that cluster 2 represents PNV cluster. The current unsupervised machine learning revealed that 46.2% of CNV belong to pachychoroid spectrum, which was higher than the proportion described in our previous report^36^. Although the prevalence of pachychoroid spectrum within CNV patients has not been sufficiently addressed in other ethnicities, at least in Japanese population, PNV is a common type of CNV.

As shown in Fig. 2, the histogram of SFCT shows a definite bimodal distribution, which represents an overlap between two normal distributions; this figure shows that CNV consists of two different diseases, PNV and conventional typical AMD, which have different choroidal structures. In the current result, the most distinguishing cut-off value of SFCT was of around 250 μm while 95% confidence interval of choroidal thickness of PNV was between 151 μm and 516 μm. Other clinical characteristics were also compatible with previous reports, which support the validity of the current clustering. For example, patients with PNV were significantly younger and showed significantly better visual acuity, while no significant difference in patients’ sex were observed between PNV and conventional typical AMD. Interestingly, axial length in patients with PNV was significantly shorter than that of patients with conventional typical AMD. As choroidal thickness generally decreases in eyes with long axial length^58–60^, this difference appears reasonable.

Large soft drusen (≥ 125 µm) are known as a risk factor for late AMD^61–63^. While patients with PNV have been reported to have less soft drusen and less pseudodrusen^28,39^, a recent report has suggested another subtype of drusen called “ pachydrusen” which is associated with thickened choroid and PNV^38^. In the present study, the frequency of “type B drusen” or “type C drusen”, reported as pachydrusen, was observed in 62.8% of all patients. This is higher than the previously reported frequency^28^. The explanation may be that (1) we diagnosed the type of drusen based solely on color fundus photographs for unbiased phenotyping, and (2) the drusen outside the macular lesion that cannot be evaluated by OCT, were counted in this study. If it was possible to evaluate all lesions by high-resolution OCT images, we could extract genuine drusen, the rate of which might be lower than the current result. Still, we successfully showed that the “type B drusen” or “type C drusen” are associated with pachychoroid spectrum as reported in the original article^28,64^. The current clustering method also demonstrated that soft drusen (Type A drusen) and pseudodrusen are rare in patients with PNV, as previously reported, suggesting that PNV is pathologically different from drusen-driven AMD.

PCV is characterized by the presence of branching vascular networks and polypoidal lesions on ICGA images, as first shown by Yannuzzi^65^. Although it has been categorized as a spectrum of conventional typical AMD, several recent papers have categorized PCV as a pachychoroid spectrum disease because (1) mean choroidal thickness in PCV is higher than that in typical AMD, and (2) the first study describing PNV reported that this pathology is often accompanied with polypoidal lesions^35^. However, in the current study, polypoidal lesions were observed more frequently in PNV (51.2%) than in conventional typical AMD (43.9%), but there was no significant difference. This result suggests that not all CNV with polypoidal lesions belong to pachychoroid spectrum. Indeed, previous studies have shown that the prevalence of soft drusen in PCV was around 23–24% in Japanese population^20,66^, meaning that a subset of PCV indeed belongs to a spectrum of conventional typical AMD. From these findings, we propose subdividing PCV into “PNV with polypoidal lesions” and “conventional typical AMD with polypoidal lesions”.

Because our system for CNV scoring is relatively simple and showed high accuracy in differentiating PNV from conventional typical AMD, it may be useful in clinical setting. Especially, the score of less than − 3.61 indicated conventional typical AMD by a positive predictive value of 100%, and the score of more than 2.63 indicate PNV by a positive predictive value of 100%. The score was significantly associated with changes in visual acuity after aflibercept fixed regimen at 3 months, while was mildly associated with that at 12 months, which can also be confirmed in Supplementary Figure 5. This may suggest that visual acuity improved faster in PNV than in conventional typical AMD after starting aflibercept treatment. A previous report has shown that the mean concentration of VEGF in aqueous humor samples was significantly lower in eyes with PNV than in those with AMD^40^. In addition, our previous report showed that the retreatment period after 3 initial ranibizumab dose was significantly longer in PNV than in conventional typical AMD^36^. Based on these results, we speculated that PNV might be less VEGF-dependent than conventional typical AMD. It could be that PNV requires a less intensive anti-VEGF therapy than conventional typical AMD. Further prospective studies, including randomized controlled study, are required to test this hypothesis. As definite diagnostic criteria are necessary to conduct a reliable prospective study, we believe that the scoring system described here will be very helpful for future analyses.

The current study presents several limitations. First, the phenotyping was subjective given that no objective measurements for recently proposed pachychoroid characteristics such as “pachyvessel,” “reduced fundus tessellation,” and “pachydrusen” have been established. Additionally, we decided the type of drusen using only color fundus photography. As the measurement of only serial horizontal OCT scans with an examination field size of 30° × 10° was routinely performed, we could not evaluate drusen that was located out of this field with OCT images. However, it is impossible to evaluate all drusen using OCT images. We think our method is reasonable to evaluate the type of drusen. Second, we excluded bilateral CNV patients from this study because we consider ophthalmic data from the unaffected eye to be important for AMD diagnosis as well. As bilateral AMD patients would have different baseline characteristics compared to unilateral AMD patients, another diagnostic model should be established to diagnose bilateral CNV patients. Third, the significant association between the CNV score and anti-VEGF treatment effect may need to be discounted because it is based on retrospective data, even though we applied an intention-to-treat approach and LOCF imputation for reducing the bias. Fourth, the current study included only Japanese subjects from a single institute hence, the validity of the score remains unclear. However, we believe that we clearly indicated the usefulness and potential clinical implications of unsupervised machine learning. The current method and its results need to be replicated for other ethnicities.

In conclusion, we demonstrated that unsupervised machine learning automatically identified that Japanese neovascular AMD consisted of two clusters, based on their clinical manifestation. The distinguishing features were not the presence of polypoidal lesion but the presence of pachychoroid-related features. Because the presence of pachychoroid-related features was significantly associated with treatment outcome, the result of the current automatic classification would be of importance for the future AMD study.

## Supplementary information

Supplementary information.

## Data Availability

The result of principal component analysis can be visualized as Supplementary data. The other datasets generated during the current study are also available from the corresponding author on reasonable request.
