# Supplementary material for: Deep phenotype unsupervised machine learning revealed the significance of pachychoroid features in etiology and visual prognosis of age-related macular degeneration
Source: Sci Rep. 2020 Oct 28;10:18423. doi: 10.1038/s41598-020-75451-5 (PMC7595218; doi:10.1038/s41598-020-75451-5)
Supplement: Supplementary file 1 — Supplementary information. [file 41598_2020_75451_MOESM1_ESM.pdf]

Supplementary Information for

Deep Phenotype Unsupervised Machine Learning Revealed the Significance of Pachychoroid Features in Etiology and Visual Prognosis of Age-related Macular Degeneration

Yoshikatsu Hosoda, MD,[1] Masahiro Miyake, MD, PhD,[1] Kenji Yamashiro, MD, PhD,[1,2] Sotaro Ooto, MD, PhD,[1] Ayako Takahashi, MD, PhD, [1] Akio Oishi, MD, PhD,[1] Manabu Miyata, MD, PhD,[1] Akihito Uji, MD, PhD,[1] Yuki Muraoka, MD, PhD,[1] Akitaka Tsujikawa, MD, PhD[1]

[1] Department of Ophthalmology and Visual Sciences, Kyoto University Graduate School of Medicine, Kyoto, Japan
[2] Department of Ophthalmology, Otsu Red-Cross Hospital, Otsu, Japan

Supplementary Table 1. Baseline parameters included in principal component and k-means analysis.

	All (N =537)	Cluster 1 (AMD-type, N = 289)	Cluster 2 (PNV-type, N =248)	P value
Mean age (years)	73.11 ± 8.43	75.03 ± 7.87	70.87 ± 8.53	< 0.0001
Sex (male: 1, female: 2)	Male: 387<br>Female: 150	Male: 208<br>Female: 81	Male: 179<br>Female: 69	1.000
Current smoking (yes: 1, no: 0)	92 (17.1 %)	51 (17.6 %)	41 (16.5 %)	0.8184
Past smoking (yes: 1, no: 0)	368 (68.5 %)	204 (70.6 %)	164 (66.1 %)	0.3053
Blinkman Index ([number of cigarettes per day] × [number of years smoking])	538.33 ± 602.75	594.26 ± 651.15	473.15 ± 534.87	0.0514
History of CSC (yes: 1, no: 0)	28 (5.2 %)	4 (1.4 %)	24 (9.7 %)	< 0.0001
History of hypertension (yes: 1, no: 0)	258 (48.0 %)	150 (51.9%)	108 (43.5 %)	0.0570
History of diabetes mellitus (yes: 1, no: 0)	77 (14.3 %)	49 (17.0 %)	28 (11.3 %)	0.0650
History of hyperlipidemia (yes: 1, no: 0)	139 (25.9 %)	81 (28.0 %)	58 (23.3 %)	0.2366
History of cerebral infarction (yes: 1, no: 0)	15 (2.8 %)	10 (3.5 %)	5 (2.0 %)	0.4325
History of myocardial infarction (yes: 1, no: 0)	13 (2.4 %)	9 (3.1 %)	4 (1.6 %)	0.3994
Lens in CNV-affected eye (phakia: 1, IOL: 0)	IOL: 94 (17.5 %)<br>Phakia: 443 (82.5 %)	IOL: 68 (23.5 %)<br>Phakia: 221 (76.5 %)	IOL: 26 (10.5 %)<br>Phakia: 222 (89.5 %)	< 0.0001
Lens in fellow eye (phakia: 1, IOL: 0)	IOL: 88 (16.4 %)	IOL: 61 (21.1 %)	IOL: 27 (10.9 %)	0.0015

	Phakia: 449 (83.4 %)	Phakia: 228 (78.9 %)	Phakia: 221 (89.1%)	
Axial length in CNV-affected eye (mm)	23.62 ± 1.09	23.83 ± 1.10	23.37 ± 1.01	< 0.0001
Axial length in the fellow eye (mm)	23.73 ± 1.11	23.97 ± 1.13	23.46 ± 1.02	< 0.0001
CVH in CNV-affected eye (yes: 1, no: 0)	93 (17.3 %)	11 (3.8 %)	82 (33.1%)	< 0.0001
CVH in the fellow eye (yes: 1, no: 0)	103 (19.2 %)	14 (4.8 %)	89 (35.9 %)	< 0.0001
logMAR BCVA in CNV-affected eye	0.322 ± 0.363	0.377 ± 0.385	0.259 ± 0.324	0.0001
logMAR BCVA in the fellow eye	-0.033 ± 0.160	-0.012 ± 0.168	-0.058 ± 0.147	< 0.0001
Polypoidal lesion in CNV-affected eye (yes: 1, no: 0)	254 (47.3 %)	127 (43.9 %)	127 (51.2 %)	0.0999
Dilated choroidal vessel in CNV-affected eye (yes: 1, no: 0)	271 (50.5 %)	70 (24.2 %)	201 (81.0 %)	< 0.0001
Dilated choroidal vessel in the fellow eye (yes: 1, no: 0)	181 (33.7 %)	28 (9.7 %)	153 (61.7 %)	< 0.0001
Geographic atrophy in CNV-affected eye (yes: 1, no: 0)	5 (0.9 %)	3 (1.0 %)	2 (0.8 %)	1.000
Geographic atrophy in the fellow eye (yes: 1, no: 0)	17 (3.2 %)	10 (3.5 %)	7 (2.8 %)	0.8064
SFCT in CNV-affected eye (μm)	259.15 ± 108.94	194.564 ± 74.187	334.42 ± 93.65	< 0.0001
SFCT in the fellow eye (μm)	255.94 ± 104.79	194.235 ± 74.29	327.85 ± 88.01	< 0.0001
RT in CNV-affected eye (μm)	402.80 ± 208.10	451.46 ± 230.42	346.11 ± 161.47	< 0.0001
RT in the fellow eye (μm)	212.51 ± 35.10	213.26 ± 33.39	211.65 ± 37.05	0.2464
GLD in CNV-affected eye (μm)	3531.57 ± 1892.63	3628.26 ± 1889.94	3418.90 ± 1893.34	0.0835
CME in CNV-affected eye (yes: 1, no: 0)	129 (24.0 %)	90 (31.1 %)	39 (15.7 %)	< 0.0001
SRH in CNV-affected eye (yes: 1, no: 0)	210 (39.1 %)	117 (40.5 %)	93 (37.5 %)	0.5348
Type 1 CNV (yes: 1, no: 0)	522 (97.2 %)	280 (96.9 %)	242 (97.6 %)	0.7942
Type 2 CNV (yes: 1, no: 0)	55 (10.2 %)	39 (13.5 %)	16 (6.5 %)	0.0097

Reduced fundus tessellation in CNV-affected eye (yes: 1, no: 0)	203 (37.8 %)	23 (8.0 %)	180 (72.6 %)	< 0.0001
Reduced fundus tessellation in the fellow eye (yes: 1, no: 0)	207 (38.5 %)	24 (8.3 %)	183 (73.8 %)	< 0.0001
Pseudodrusen in CNV-affected eye (yes: 1, no: 0)	16 (3.0 %)	16 (5.5 %)	0 (0 %)	< 0.0001
Pseudodrusen in the fellow eye (yes: 1, no: 0)	29 (5.4 %)	28 (9.7 %)	1 (0.4 %)	< 0.0001
Drusen A				
Within 6mm from macular lesion				
Number of drusen in CNV-affected eye (N)	0.080 ± 0.344	0.128 ± 0.418	0.024 ± 0.219	< 0.0001
Number of drusen in the fellow eye (N)	0.186 ± 0.464	0.301 ± 0.549	0.052 ± 0.287	< 0.0001
Size of drusen in CNV- affected eye (size of the largest drusen cluster)	0.111 ± 0.551	0.196 ± 0.735	0.012 ± 0.100	< 0.0001
Size of drusen in the fellow eye (size of the largest drusen cluster)	0.287 ± 0.782	0.481 ± 0.980	0.060 ± 0.334	< 0.0001
Outside macular lesion				
Number of drusen in CNV-affected eye (N)	0.030 ± 0.235	0.052 ± 0.313	0.004 ± 0.064	0.0203
Number of drusen in the fellow eye (N)	0.035 ± 0.260	0.055 ± 0.329	0.012 ± 0.142	0.0383
Size of drusen in CNV-affected eye	0.043 ± 0.363	0.076 ± 0.489	0.004 ± 0.064	0.0203
Size of drusen in fellow eye	0.036 ± 0.343	0.064 ± 0.464	0.004 ± 0.045	0.0372
Drusen B				
Within 6mm from macular lesion				
Number of drusen in CNV-affected eye (N)	0.488 ± 0.941	0.211 ± 0.578	0.810 ± 1.156	< 0.0001
Number of drusen in the fellow eye (N)	0.665 ± 1.051	0.325 ± 0.720	1.060 ± 1.224	< 0.0001
Size of drusen in CNV-affected eye	0.293 ± 0.743	0.114 ± 0.357	0.502 ± 0.983	< 0.0001
Size of drusen in the fellow eye	0.391 ± 0.800	0.173 ± 0.465	0.645 ± 1.009	< 0.0001

Outside macular lesion				
Number of drusen in CNV-affected eye (N)	0.274 ± 0.650	0.135 ± 0.416	0.435 ± 0.817	< 0.0001
Number of drusen in the fellow eye (N)	0.259 ± 0.584	0.152 ± 0.422	0.383 ± 0.710	< 0.0001
Size of drusen in CNV-affected eye	0.176 ± 0.542	0.080 ± 0.290	0.288 ± 0.719	< 0.0001
Size of drusen in the fellow eye	0.177 ± 0.497	0.090 ± 0.268	0.278 ± 0.658	< 0.0001
Drusen C				
Within 6mm from macular lesion				
Number of drusen in CNV-affected eye (N)	0.210 ± 0.535	0.111 ± 0.384	0.327 ± 0.651	< 0.0001
Number of drusen in the fellow eye (N)	0.300 ± 0.673	0.135 ± 0.432	0.492 ± 0.834	< 0.0001
Size of drusen in CNV-affected eye	0.151 ± 0.416	0.083 ± 0.306	0.230 ± 0.505	< 0.0001
Size of drusen in the fellow eye	0.173 ± 0.399	0.078 ± 0.246	0.284 ± 0.502	< 0.0001
Outside macular lesion				
Number of drusen in CNV-affected eye (N)	0.043 ± 0.229	0.0173 ± 0.1549	0.0726 ± 0.289	0.0021
Number of drusen in the fellow eye (N)	0.039 ± 0.229	0.0138 ± 0.117	0.0685 ± 0.311	0.0105
Size of drusen in CNV-affected eye	0.040 ± 0.242	0.01384 ± 0.1242	0.0706 ± 0.3276	0.0020
Size of drusen in the fellow eye	0.030 ± 0.200	0.0103 ± 0.0926	0.0524 ± 0.276	0.0108

CSC: central serous chorioretinopathy, MAR: minimum angle resolution, BCVA: best-corrected visual acuity, CNV: choroidal neovascularization, CVH: choroidal vascular hyperpermeability, IOL: intra ocular lens. SFCT: subfoveal choroidal thickness, RT: retinal thickness, GLD: greatest linear dimension, CME: cystoid macular edema, SRH: subretinal hemorrhage.

Supplementary Table 2. Diagnostic protocols for baseline characteristics.

Phenotype	Phenotyping protocol	Initially described
Choroidal vascular hyperpermeability	Choroidal vascular hyperpermeability was defined as a multifocal area of hyperfluorescence with blurred margins in late-phase indocyanine green angiography images.[1–3]	1994
Polypoidal lesion	Peculiar polypoidal, subretinal, vascular lesions in indocyanine green angiography images.[4–7]	1990
Dilated choroidal vessel	Dilated choroidal vessel was defined as focal choriocapillaris thinning with focal inward displacement of large choroidal vessels in OCT images.[8–11]	2014
Subfoveal choroidal thickness	Choroidal thickness was defined as the vertical distance between the Bruch membrane and the hyper-reflective line behind the large choroidal vessel layers (subfoveal choroidal-scleral interface) using enhanced depth OCT imaging.[12–14]	2008
Geographic atrophy	Geographic atrophy was defined as oval areas of atrophy of the RPE and choriocapillaris.[15–17]	1975
Retinal thickness	Retinal thickness was defined as the distance between the vitreoretinal interface and the Bruch membrane.[18–20]	1995
Greatest linear dimension	The greatest linear dimension of a lesion was determined by measuring the entire CNV with fluorescein angiography. Contiguous RPE detachment or subretinal hemorrhage were included in the lesion according to the Treatment of Age-related macular degeneration with Photodynamic therapy Study protocol.[21]	1999
Cystoid macular edema	Cystoid macular edema was defined as retinal thickening of the macula, diagnosed from the depiction of intraretinal cystic areas of low reflectivity in OCT images.[18,22]	1991
Subretinal hemorrhage	Subretinal hemorrhage was defined as an accumulation of blood between the neurosensory retina and the RPE using color fundus photographs and OCT images.	1948
Type of CNV	CNV located beneath the retinal pigment epithelium was defined as type 1; CNV between the sensory retina and retinal pigment epithelium was defined as type 2; the classification was based on	1994

	OCT images.[23,24]	
Reduced fundus tessellation	Reduced fundus tessellation was defined as a focal, multifocal, or diffuse area of reddish orange background within the arcades with minimal to absent choroidal vascular markings.[25]	2013
Pseudodrusen	Pseudodrusen was defined as extracellular accumulations of material inside the RPE, under the retina.[26–28]	1990
Type A drusen (Soft drusen)	Type A drusen was defined as homogeneous sub-RPE deposits forming mounds corresponding to yellow-white aggregates seen in color photography, according to the Age-Related Eye Disease Study.[29,30]	1991
Type B drusen	Type B drusen typically have complex outer contour. The choroid seems featureless and has a redder hue than thinner choroids.[30]	2018
Type C drusen	Type C drusen have an undercut, eroded outer contour. There can be projections jutting from the central accumulations of material.[30]	2018
CNV: choroidal neovascularization, OCT: optical coherence tomography, RPE: retinal pigment epithelium.		

Supplementary Table 3. The results of gap statistics analysis.

Number of clusters (*k*)	Log (W_k)	E.log (W_k)	Gap	Standard error
1	7.242881	8.178464	0.9355830	0.003778170
2	7.207623	8.156720	0.9490963	0.006066069
3	7.191034	8.141124	0.9500909	0.006464777
4	7.172879	8.128467	0.9555876	0.006592169
5	7.172879	8.118256	0.9648880	0.005734980
W_k: the sum of the pairwise distance for all points upon clustering the dataset into k clusters. E.log (W_k): expectation value of log (W_k) for an appropriate null reference distribution of the data.				

Supplementary Figure 1. Flow diagram of the participant selection process. FA = fluorescein angiography; **ICGA** = indocyanin green angiography; **OCT** = optical coherence tomography; **EDI** = enhanced depth imaging.

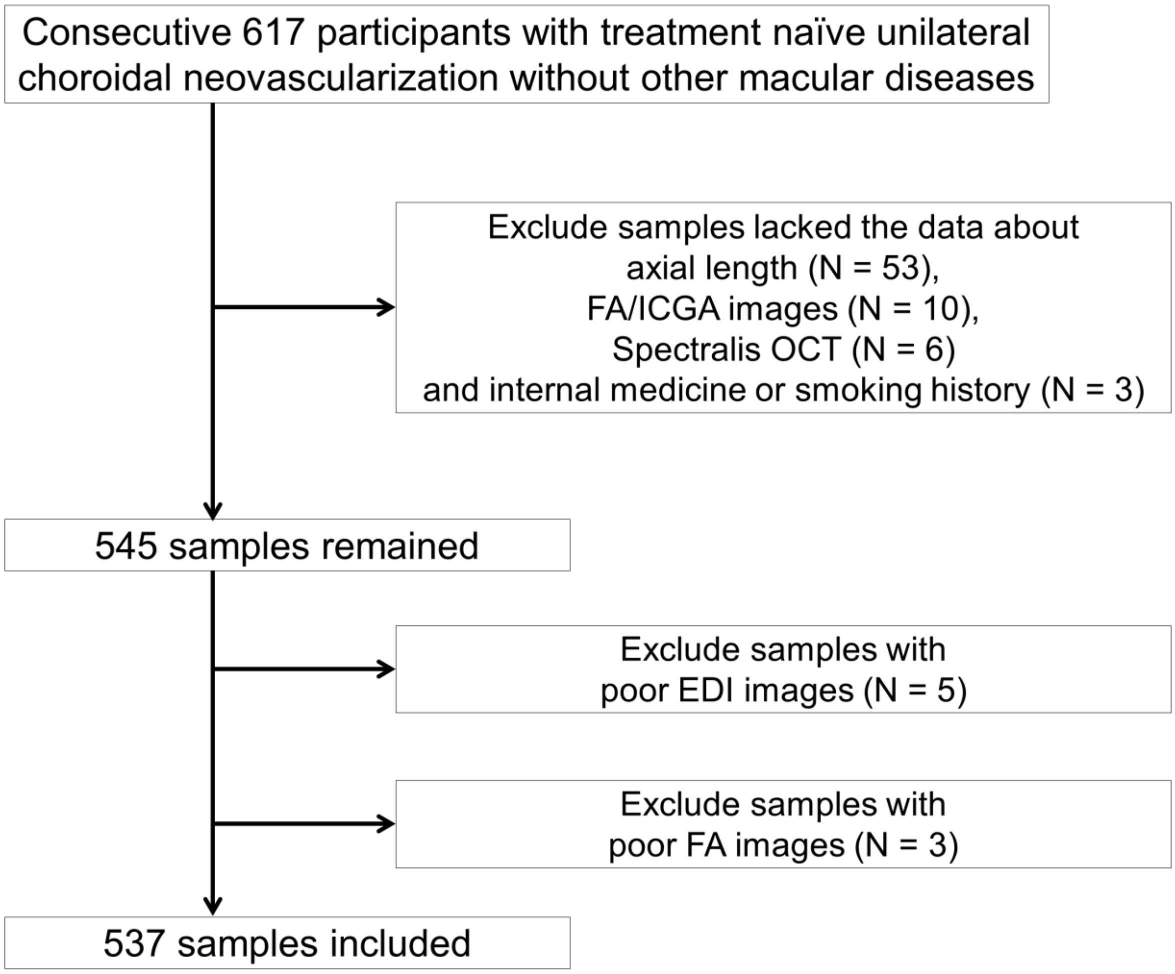

Supplementary Figure 2. Line graph showing gap and numbers of clusters. Error bars depict a standard error for gap values.

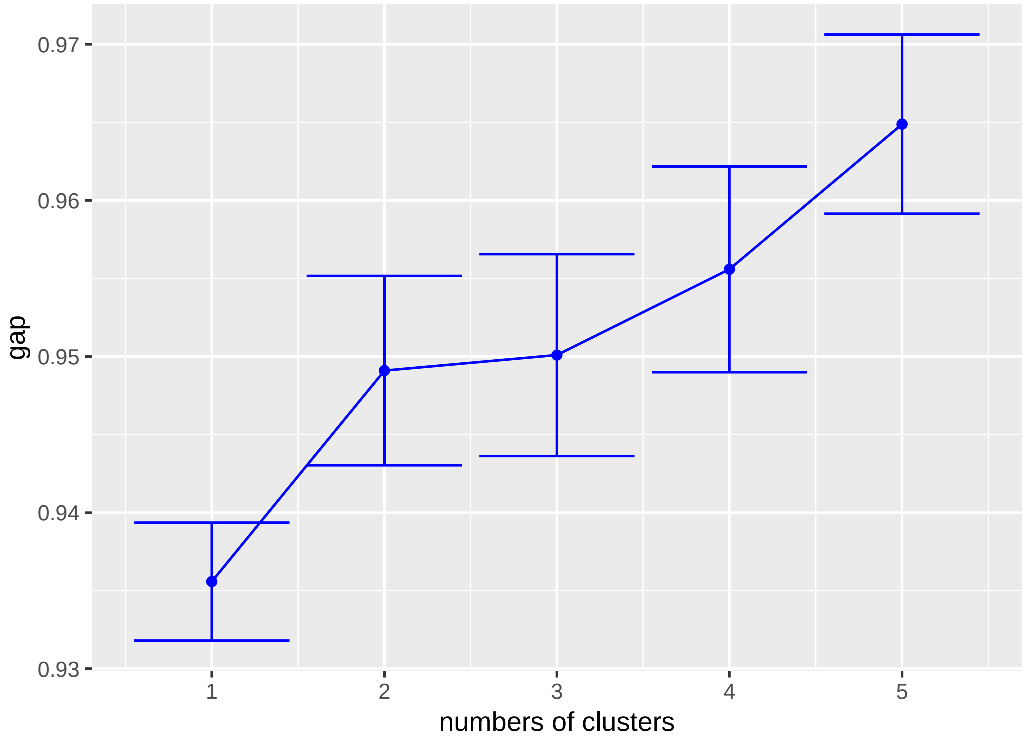

Supplementary Figure 3. A case of patient who met our previous PNV diagnostic criteria, and classified into AMD-type cluster

A 71-year-old male patient was visually impaired in the left eye. Images from the right eye (A to E) and left eye (F to J). (A) Color fundus photograph shows no CNV. (B) Fluorescein angiography image shows no leakage. (C) Late phase of indocyanine green angiography image shows no choroidal vascular hyperpermeability spots. Choroidal vascular hyperpermeability spots were also observed around the macular region. (D) Infrared reflectance image of right eye. (E) Foveal vertical EDI OCT scan shows mildly thickened choroid. Subfoveal choroidal thickness was 258 μm and retinal thickness was 210 μm. (F) Color fundus photograph shows serous retinal detachment within macular region. (G) Fluorescein angiography image shows leakage within the region of CNV. (H) Late phase of indocyanine green angiography image shows a polypoidal lesion (blue arrow). (I) Infrared reflectance image of left eye. (J) Foveal horizontal EDI OCT scan shows mildly thickened choroid and dilated choroidal vessels (blue arrows). Subfoveal choroidal thickness was 228 μm and retinal thickness was 410 μm.

CNV; choroidal neovascularization. EDI; enhanced depth imaging. OCT; optical coherence tomography. PNV; pachychoroid neovasculopathy.

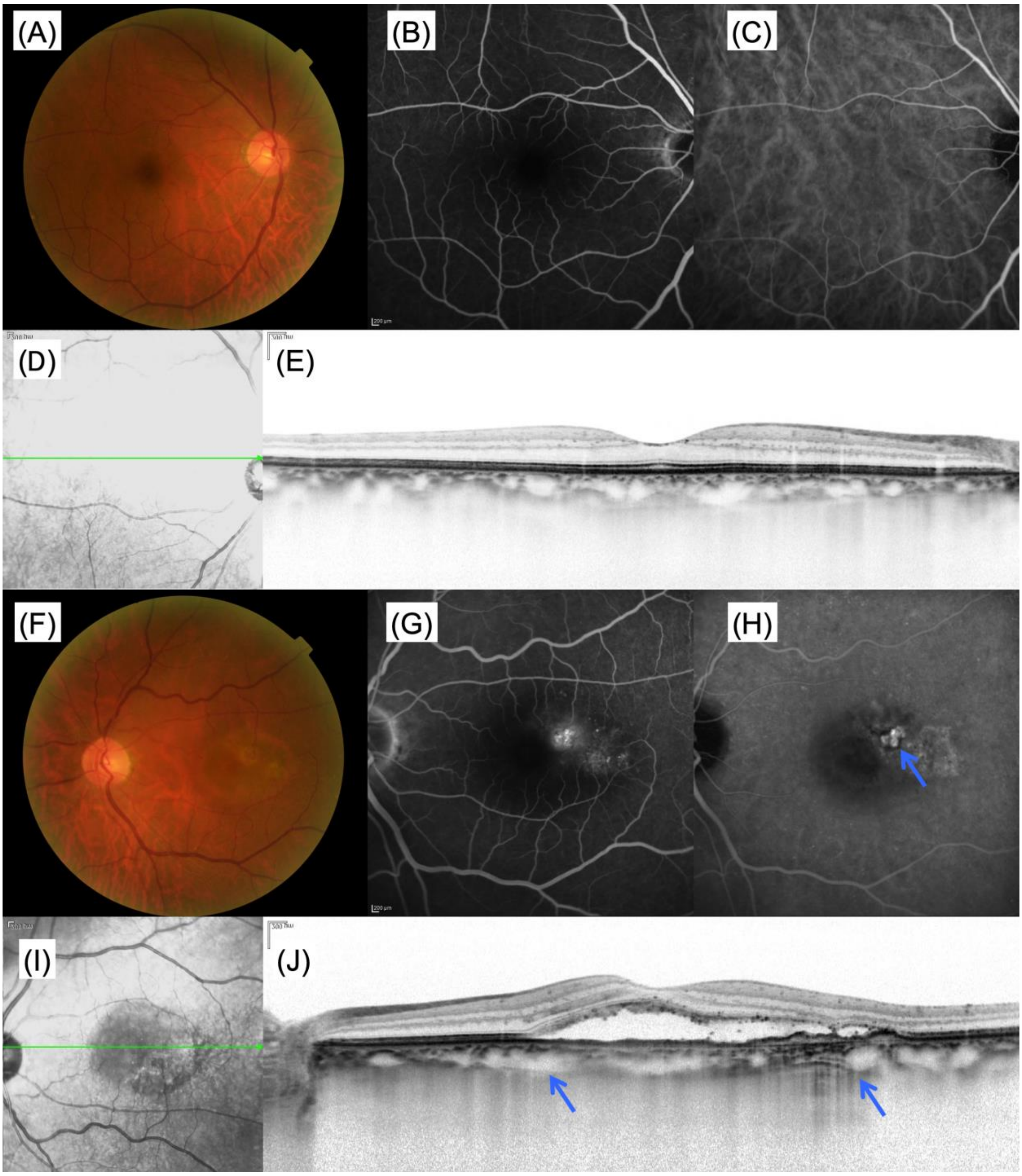
(A)
(B)
(C)
(D)
(E)
(F)
(G)
(H)
(I)
(J)

Supplementary Figure 4. ROC curves representing the results of CNV scoring system applied to validation and total datasets.

(A) ROC curve derived from validation dataset (AUC = 0.939). (B) ROC curve derived from the total dataset (AUC = 0.938).

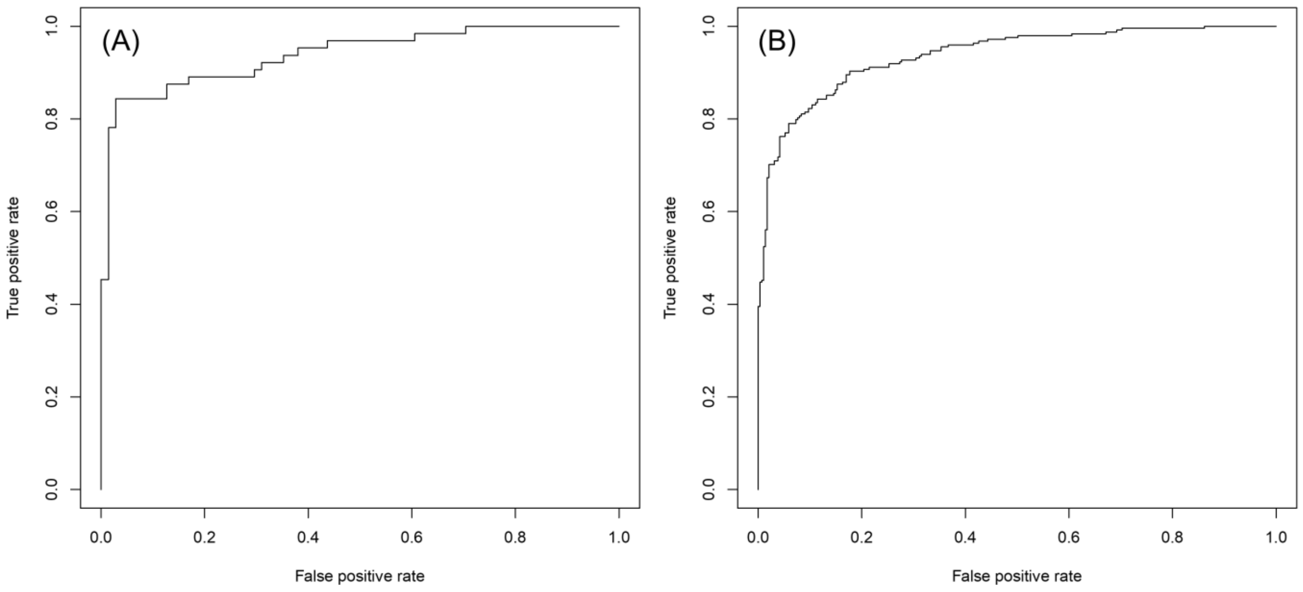

Supplementary Figure 5. Time course changes in logMAR BCVA in AMD-type and PNV-type patients treated with aflibercept

Time course changes of logMAR BCVA in patients treated with aflibercept injections. Eyes in PNV-type cluster (N=126) showed relatively rapid improvement in visual acuity (LogMAR at baseline: 0.231 ±.0.284, at 3 months: 0.177 ± 0.304, at 12 months: 0.161 ± 0.357) compared to the eyes in AMD-type cluster (N=154, LogMAR at baseline: 0.355 ±.0.356, at 3 months: 0.323 ± 0.378, at 12 months: 0.275 ± 0.373). All values are shown as mean ± standard deviation. Error bars depict standard deviation from the mean.

MAR; minimum angle resolution, BCVA; best corrected visual acuity, SD; standard deviation, AMD; age-related macular degeneration, PNV; pachychoroid neovasculopathy.

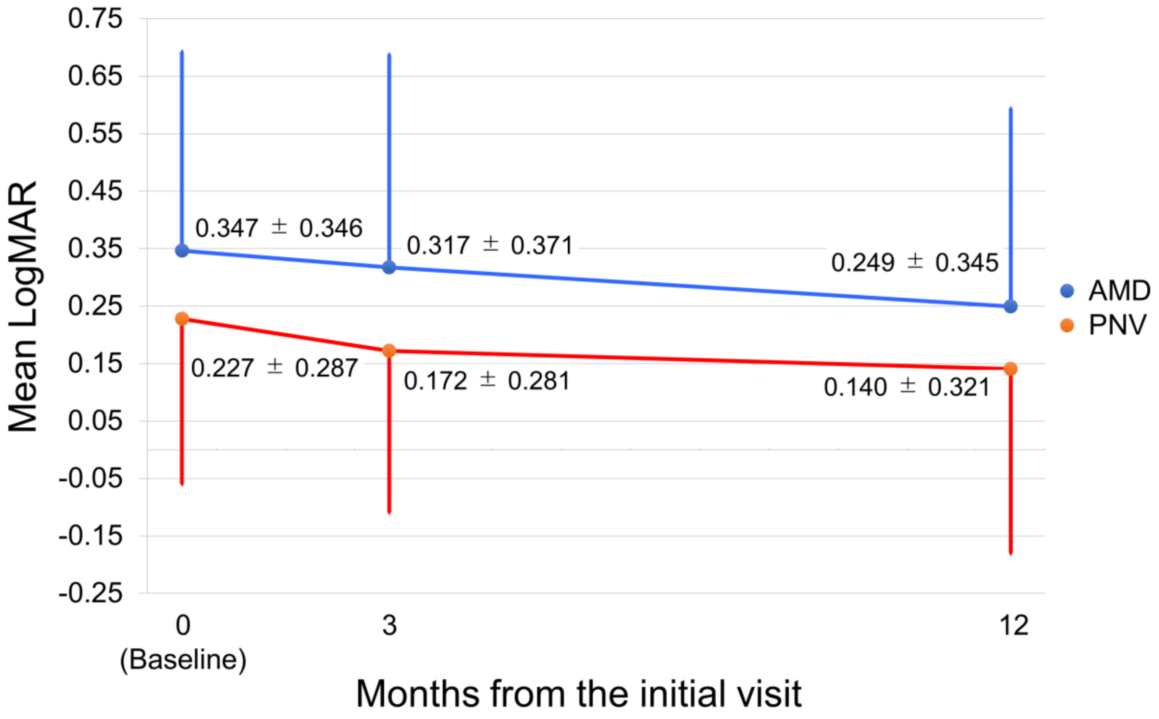

References

1. Peyman, G. A. Choroidal Hyperpermeability in Central Serous Choroidopathy: A New Concept? *Archives of Ophthalmology* **113**, 701–702 (1995).
2. Piccolino, F. C. & Borgia, L. Central serous chorioretinopathy and indocyanine green angiography. *Retina* **14**, 231–242 (1994).
3. Guyer, D. R. *et al.* Digital Indocyanine Green Videoangiography of Central Serous Chorioretinopathy. *Arch. Ophthalmol.* **112**, 1057–1062 (1994).
4. Yannuzzi, L. A., Sorenson, J., Spaide, R. F. & Lipson, B. Idiopathic polypoidal choroidal vasculopathy (IPCV). *Retina* **10**, 1–8 (1990).
5. Yannuzzi, L. A. *et al.* The expanding clinical spectrum of idiopathic polypoidal choroidal vasculopathy. *Arch. Ophthalmol.* **115**, 478–485 (1997).
6. Phillips, W. B., Regillo, C. D. & Maguire, J. I. Indocyanine green angiography of idiopathic polypoidal choroidal vasculopathy. *Ophthalmic Surg. Lasers* **27**, 467–470 (1996).
7. Spaide, R. F., Yannuzzi, L. A., Slakter, J. S., Sorenson, J. & Orlach, D. A. Indocyanine green videoangiography of idiopathic polypoidal choroidal vasculopathy. *Retina* **15**, 100–110 (1995).

8. Azar, G. *et al.* Pachychoroid neovasculopathy: aspect on optical coherence tomography angiography. *Acta Ophthalmol.* **95**, 421–427 (2017).

9. Dansingani, K. K., Balaratnasingam, C., Naysan, J. & Freund, K. B. En face imaging of pachychoroid spectrum disorders with swept-source optical coherence tomography. *Retina* **36**, 499–516 (2016).

10. Gallego-Pinazo, R., Dolz-Marco, R., Gómez-Ulla, F., Mrejen, S. & Freund, K. B. Pachychoroid diseases of the macula. *Med. hypothesis, Discov. Innov. Ophthalmol. J.* **3**, 111–115 (2014).

11. Pang, C. E. & Freund, K. B. Pachychoroid neovasculopathy. *Retina* **35**, 1–9 (2015).

12. Ikuno, Y., Kawaguchi, K., Nouchi, T. & Yasuno, Y. Choroidal thickness in healthy Japanese subjects. *Investig. Ophthalmol. Vis. Sci.* **51**, 2173–2176 (2010).

13. Margolis, R. & Spaide, R. F. A Pilot Study of Enhanced Depth Imaging Optical Coherence Tomography of the Choroid in Normal Eyes. *Am. J. Ophthalmol.* **147**, 811–815 (2009).

14. Spaide, R. F., Koizumi, H. & Pozonni, M. C. Enhanced Depth Imaging Spectral-Domain Optical Coherence Tomography. *Am. J. Ophthalmol.* **146**, 496–500 (2008).

15. Schmitz-Valckenberg, S. *et al.* Geographic atrophy: Semantic considerations and literature review. *Retina* **36**, 2250–2264 (2016).

16. Willerson, D. & Aaberg, T. M. Senile macular degeneration and geographic atrophy of the retinal pigment epithelium. *Br. J. Ophthalmol.* **62**, 551–553 (1978).

17. Blair, C. J. Geographic Atrophy of the Retinal Pigment Epithelium: A Manifestation of Senile Macular Degeneration. *Arch. Ophthalmol.* **93**, 19–25 (1975).

18. Hee, M. R. *et al.* Quantitative Assessment of Macular Edema With Optical Coherence Tomography. *Arch. Ophthalmol.* **113**, 1019–1029 (1995).

19. Hee, M. R. *et al.* Optical coherence tomography of age-related macular degeneration and choroidal neovascularization. *Ophthalmology* **103**, 1260–1270 (1996).

20. Hee, M. R. *et al.* Topography of diabetic macular edema with optical coherence tomography. *Ophthalmology* **105**, 360–370 (1998).

21. Bressler, N. M. Photodynamic therapy of subfoveal choroidal neovascularization in age- related macular degeneration with verteporfin: One-year results of 2 randomized clinical trials - TAP report 1. *Arch. Ophthalmol.* **117**, 1329–1345 (1999).

22. Huang, D. *et al.* Optical coherence tomography. *Science (80-.).* **254**, 1178–1181 (1991).

23. Grossniklaus, H. E. & Gass, J. D. M. Clinicopathologic correlations of surgically excised type 1 and type 2 submacular choroidal neovascular membranes. *Am. J. Ophthalmol.* **126**, 59–69 (1998).

24. Gass, J. D. M. Biomicroscopic and histopathologic considerations regarding the feasibility of surgical excision of subfoveal neovascular membranes. *Am. J. Ophthalmol.* **118**, 285–298 (1994).

25. Warrow, D. J., Hoang, Q. V. & Freund, K. B. Pachychoroid pigment epitheliopathy. *Retina* **33**, 1659–1672 (2013).

26. Mimoun, G., Soubrane, G. & Coscas, G. Macular drusen. *Journal Francais d'Ophtalmologie* **13**, 511–530 (1990).

27. Maguire, M. G. & Fine, S. L. Reticular pseudodrusen. *Retina* **16**, 167–8 (1996).

28. Arnold, J. J., Sarks, S. H., Killingsworth, M. C. & Sarks, J. P. Reticular pseudodrusen: A risk factor in age-related maculopathy. *Retina* **15**, 183–191 (1995).

29. Klein, R. *et al.* The Wisconsin Age-related Maculopathy Grading System. *Ophthalmology* **98**, 1128–1134 (1991).

30. Spaide, R. F. DISEASE EXPRESSION in NONEXUDATIVE AGE-RELATED MACULAR DEGENERATION VARIES with CHOROIDAL THICKNESS. *Retina*

38, 708–716 (2018).
